# The use of microblog-based case studies in a pharmacotherapy introduction class in China

**DOI:** 10.1186/1472-6920-13-120

**Published:** 2013-09-08

**Authors:** Tiansheng Wang, Fei Wang, Luwen Shi

**Affiliations:** 1 Department of Pharmacy Administration and Clinical Pharmacy, Peking University Health Science Center, Beijing, China; 2 Department of Pharmacy Practice, School of Pharmacy, University of Connecticut, Hartford, Connecticut, USA

## Abstract

**Background:**

Microblog is a Web 2.0 technology that provides an online social networking platform for communicating and sharing information among web users. Pharmacy educators have previously used microblog to promote active engagement of students. However, there is very little research to demonstrate how to use microblogging effectively to enhance pedagogy in a blended or face-to-face classroom environment. We used the most popular microblog website in China to create a “space” within the classroom to evaluate an interactive microblogging forum for the integration of pharmacotherapy case studies. This study is aimed to determine students’ attitudes toward microblog-based case studies (MBC) in a pharmacotherapy class.

**Methods:**

We created a group on Sina Weibo, the most popular microblog website in China, to explore the possibilities of using microblog-based case discussions in pharmacy education to promote and motivate student learning. The class teaching activities began in November 2011; individual group assignments to a single case study were administered to 21 groups with a total of 126 participating pharmacy students. Each group was required to share a discussion care plan on the microblogging platform. Individual students were expected to participate in an online discussion related to at least two other group cases by posting their comments on the microblog platform. All postings were tracked and analyzed, and then a post MBC survey was administered anonymously to determine students’ opinions towards MBC.

**Results:**

A total of 126 students posted 592 messages and 112 students (89%) completed the survey. More than 80% of students agreed that MBC improved communication; nearly 70% agreed that MBC increased the amount of interaction, and over 50% found value in reading other students’ messages. However, 25% students believed the collaborative learning was not effective and 22% indicated the quality of interaction was low.

**Conclusions:**

MBC appears to be well-accepted learning method to students in this study. Educators who wish to use MBC for pharmacy courses should balance the potential advantages, such as improving communication and the amount of interaction, with potential disadvantages, such as inefficient collaborative learning and the low quality of interaction.

## Background

Today’s college students between the ages of 18 and 24 belong to the next generation [1], they are educated in a computer-technology-based environment and prefer active learning methods using technology and collaborative teamwork [2]. Studies have shown that Web 2.0 applications (e.g., blogs, microblogging, video and photo sharing, social media and social bookmarking) allow students to express their ideas for others to read, encourage an open dialogue and increase interaction among users [3-5].

Microblog is a Web 2.0 technology that provides an online social networking platform for communicating and sharing information among web users. A microblog differs from a blog in that it has smaller amount of space for writing content: 140 characters or less per post, similar to a mobile text message. Users can join the service free of charge and send and receive short messages via the web, SMS, instant messaging clients and by third party applications using mobile technologies or computers [6]. Twitter, the most popular microblogging platform in the U.S. has application in many domains, including media outlets, politics, health-care, public health and education [7-13]. In recent years, microblogging has been adopted in different educational settings for a variety of educational purposes [14]. Current evidence suggests that microblogging has the potential to encourage student participation, engagement, reflective thinking and collaborative learning [15]. Microblogging can solidify and enhance in-class face-to-face learning by continuing the conversation outside the classroom walls for a more sustained learning experience [14,16]. It is a powerful way to create a strong learning community that involves all students, especially those who are shy or less outspoken in a face-to-face class [17].

In a recent survey of Colleges of Pharmacy in the U.S., the use of blogging/microblogging to engage students was 59.5% [18]. A recent study reported the use of microblogging in an on-line course [19], but there is very little research to demonstrate how to use microblogging effectively to enhance pedagogy in a blended or face-to-face classroom environment.

Since the largest microblogging website in China is Sina Weibo [20] (Weibo, weibo.com, the Chinese version of Twitter) and about 46% of students are microblog users [21], we used Weibo to create a “space” within the classroom to evaluate an interactive microblogging forum for the integration of pharmacotherapy case studies. A preliminary survey to explore pharmacy student usage of microblogging demonstrated that 24 (22%) use microblog every day, 50 (45%) use several times a week, 29 (26%) reported never used before. The research aim of this study was to determine pharmacy students’ attitudes towards MBC. Students’ microblog activity and a post-activity student survey were used to determine the students’ level of engagement and perception of their experience. This research was approved by the Institutional Review Board of the School of Pharmaceutical Sciences, Peking University.

## Methods

### Pharmacy students and the pharmacotherapy introduction class

A total of 126 pharmacy students in the 4th year of a 6-year continuous Master of Science program [22] at Peking University were enrolled in a pharmacotherapy introduction course. The 17-week course met once weekly for 3 hours. This required course was designed to provide introductory information to students and assist them to understand the rationale upon which many therapy decisions are based. Principles, concepts, processes, and skills in pharmacotherapy were emphasized; case studies were used to provide students with the opportunity to apply these skills. Therapeutic topics covered included an introduction to respiratory, cardiovascular, gastrointestinal, endocrine, hematology, oncology, neurology, and renal disease. This is a team taught course and the 3 classes (week 12,13,14) with MBC were taught by the author T.W. Fifteen minutes class time were devoted to teach students who were not current Weibo users how to sign up for a Weibo account, send tweets, use @replies, and upload an image, etc.

As educators must address issues concerning privacy and professionalism when using social networking sites [23,24], we created a closed Weibo group and recommended students register new usernames for this assignment. The instructor and graduate students had access to these registered usernames to track student posts. All comments were required to be posted in an appropriate and professional manner.

### Survey instrument

An anonymous survey questionnaire was administered in class to all students following completion of all case discussions and presentations in week 14. Participation in the survey was voluntary and did not affect final student grades. There were 8 questions addressing demographic information to put the data into context.

The survey also included 12 validated questions [25,26] which were designed to address the following aspects of MBC: perceived learning (question 1–7), sense of community (question 8–11), and satisfaction (question 12). All questions were reviewed for face validity by 3 pharmacy faculty members. Participants rated each item on a five-point Likert-type scale: strongly agree, agree, neutral, disagree, and strongly disagree. Frequency statistics were calculated for each question.

In addition, there were 2 open-ended questions to give students an opportunity to comment on their likes and dislikes of using MBC. The authors read each response to the open-ended questions and organized the data into categories based on emerging themes. A response was categorized in multiple categories when covering more than one theme. All responses and categories were reviewed by a different faculty member for agreement; discussions ensued to determine the best, agreed-upon categorization when disagreement existed.

### Microblog-based case studies

**Figure 1:**
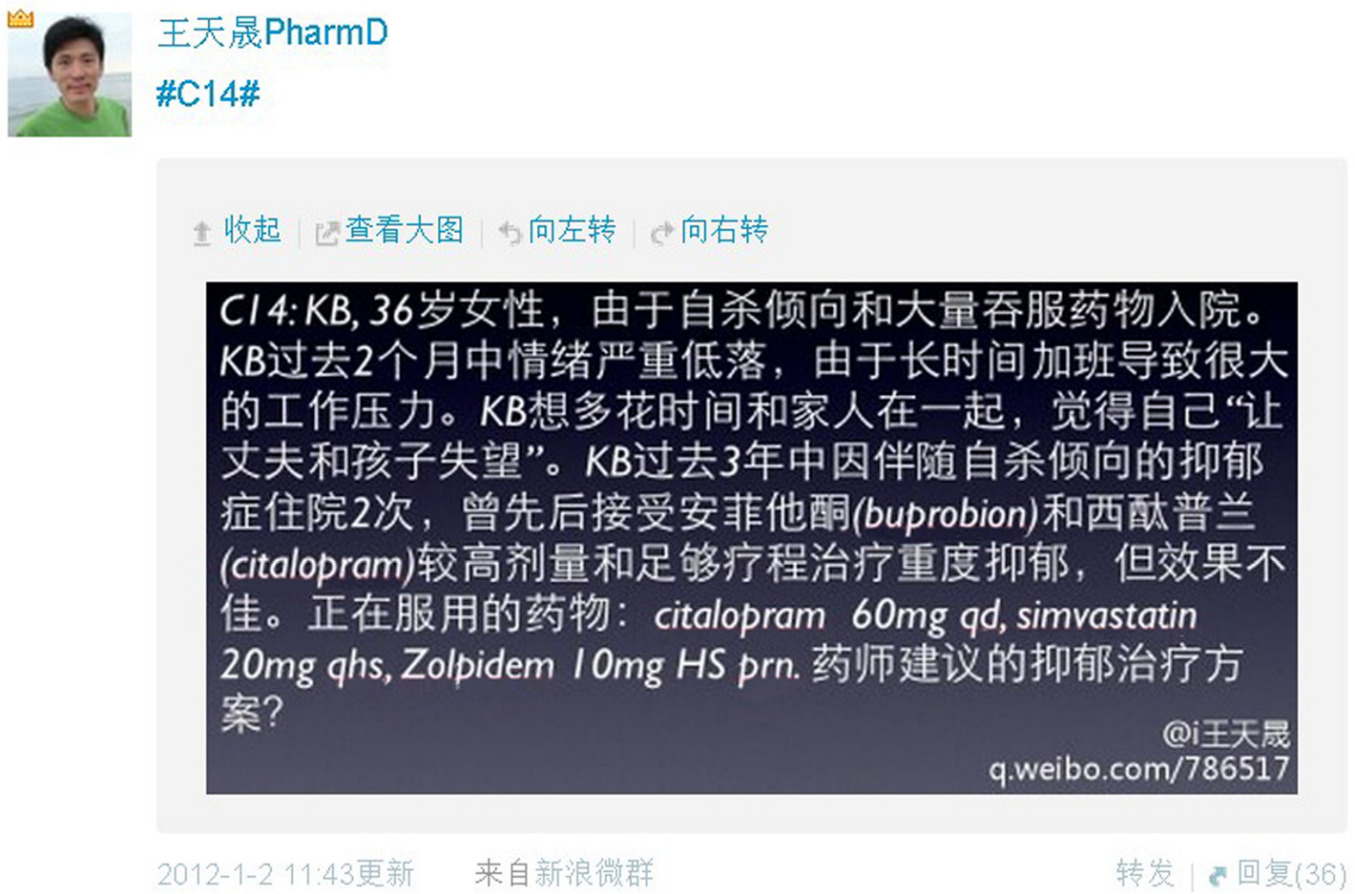
A case posted on Weibo by the instructor.

The 126 students were divided into 21 groups of 6 students each. A series of 21 different cases (i.e. mini-assignments or questions based upon one major patient case) was developed by the instructor in weeks 12 and 13 and one case was assigned to each of the 21 groups; cases closely paralleled the lectures presented in the didactic portion of the course and covered pain management (case 1–10) and depression (case 11–21). The 21 cases focused on key issues related to therapeutics, selection of appropriate drug therapy, monitoring parameters for changes in therapy, and provision of patient counseling information. As the 21 cases were designed for a pharmacotherapy introduction class, the level of complexity was simple (see Appendix 1 for an example). A case posted on Weibo is shown in Figure 1, and the format of the pharmaceutical care plan is provided in Table 1.

**Table 1:** The format of pharmaceutical care plan

Findings	Therapeutic goals & desired endpoint	Recommendations	Monitoring parameters	Patient education

All cases were required to be completed within 1 week. The 21 cases were posted on Weibo on class Day 1, the cases were to be assessed first by each individual within the group and then each group was asked to work together to provide a pharmaceutical care plan on Days 2 and 3. The care plan was developed in PowerPoint software, which was then saved as an image and uploaded to Weibo on day 4; and the image was also then presented in class.

Then students were required to read other groups’ cases and post comments (at least 2 per individual) from Day 4 to Day 7. When participating in the online discussion, students were required to integrate and apply knowledge and skills learned in the curriculum to the case studies. Comments were expected to be written in a structured, insightful way to reflect students’ learning, critical thinking, and problem-solving skills. Students were supposed to comment on the following aspects of the pharmacotherapy plan: (1) disease state information, (2) therapeutic goals, (3) drug information, (4) adverse drug events, (5) drug interactions, (6) monitoring plan, and (7) patient education. Instructions and examples were provided in class by the instructor.

The 126 students were encouraged to study more than one case and post as many times as they wanted as long as they remained on the cases; and they could use any device (PC, smartphone, iPad, etc.) to post comments. From class Day 1 to the day before the following class (Day 7), 2 graduate students were responsible for creating an archive of all comments and tabulating the number of comments by each student. The instructor started reading each post on Day 4 and answered questions when receiving @comment from students. The following class (week 14) was devoted to case presentations and discussions: each group had one student present the case and the pharmaceutical care plan, and then the instructor asked questions and further addressed the knowledge point to grade the assignments.

### Analysis of the interactivity of online discussions

An analysis of the content of text-based discussions can be utilized to evaluate whether or not effective learning occurred and to judge the group collaborative process versus the individual’s contribution to that process [27]. There is currently no standardized method to analyze the quality of online interactions in formal educational settings [27]. Our study utilized Henri’s analytical model [28] which has been widely used by educators to study the learning process of computer-mediated discussion groups in formal educational settings. A central concept adopted in the content analysis instrument is interactivity, which is stated as a three-step process: communication of information, a first response to this information, and a second answer relating to the first [27]. Henri’s analytical model consists of 5 dimensions: a participative, social, interactive, cognitive, and metacognitive dimension [28]. The model provides a basic theoretical framework for this study: using this model, 4 dimensions of MBC were examined: (1) participation, (2) social presence, (3) interaction, and (4) cognitive skills. The metacognitive dimension was omitted because no metacognitive activity was noticed in MBC.

#### Participation

The levels and types of participation were assessed by the number of daily messages exchanged between students and between students and instructor and the total number of messages for each of the 21 cases.

#### Social presence

Henri’s content analysis framework defined “social” as a “statement or part of a statement not related to the formal content of the subject matter” [28]. Thus messages not related to the pharmaceutical care plan (e.g., greetings and expression of feelings) in MBC were counted as social cues.

#### Interactivity

Interactivity (or chain of connected messages) was assessed by measuring direct comment (comment to the 21 pharmaceutical care plans), reply to comment (reply to direct comments, which contains the sign @), and @comment (used when a student wants to address a specific person). Messages were used as indicators of those interactions. Learner-content interaction was indicated by the direct comments. Learner-learner interaction was indicated by student @comments to other students and by reply to comments. Learner-instructor interaction was indicated by @comments to the instructor and other comments occurring between the students and the instructor.

#### Cognitive skills

To analyze the quality of messages and level of critical thinking, the students’ messages were categorized into 5 different levels: elementary clarification, in-depth clarification, inference, judgment, and application of strategies. All responses and categories were reviewed by 2 faculty members for agreements, discussions ensued to determine the best, agreed-upon categorization when disagreement existed.

## Results

### Analysis of microblog posts

**Figure 2:**
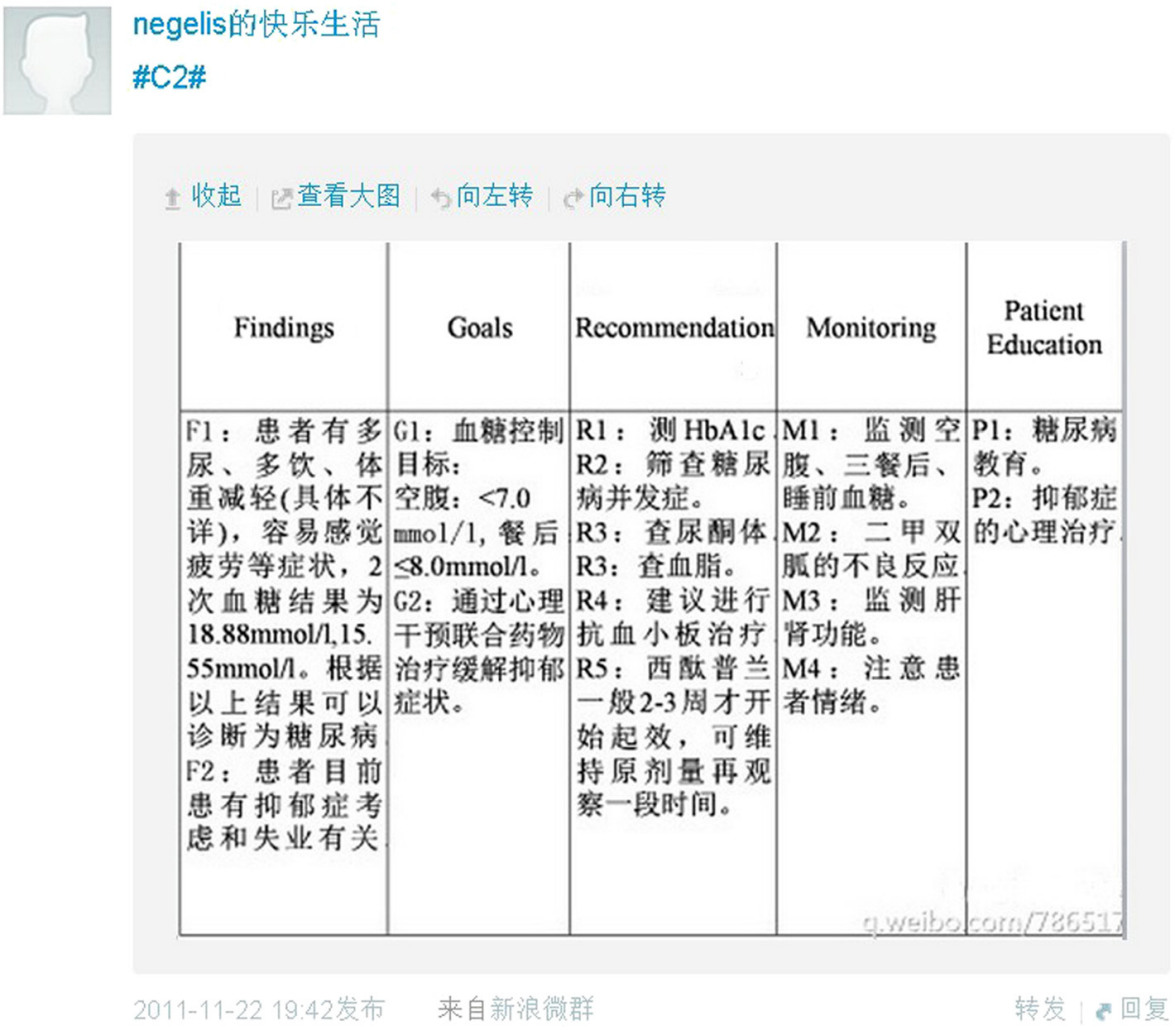
An image of pharmaceutical care plan posted on Weibo.

**Figure 3:**
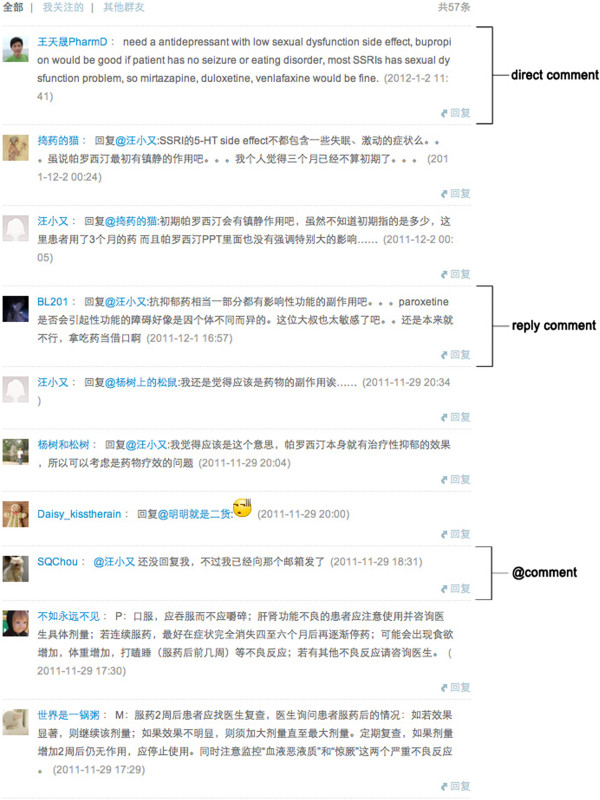
An image of online discussion on Weibo platform.

An image of a pharmaceutical care plan posted on Weibo is shown in Figure 2 and an image of online discussions is shown in Figure 3. One hundred and twenty-six students posted 592 messages during the 7 days in which their posts were recorded (Table 2). The average number of postings for each student was about 5, 13 (10%) students did not meet the minimum of 2 postings. No inappropriate posts were identified. As the class was on Friday (Day 1) and Days 2 and 3 fell on the weekend, no messages were posted until the following Monday (Day 4); and students posted most frequently on Day 5. The number of messages for each case was recorded in Table 3: assigned groups contributed more tweets towards their own case except in groups 12, 13, 16, and 21.

**Table 2:** Messages by students and instructor

**Day**	**User**	
	**Students**	**Instructor**
1	0	0
2	0	0
3	0	0
4	176	8
5	274	14
6	52	4
7	90	11
Total	592	37

**Table 3:** Message number for each case

**Group**	**Message number for each case (Case 1 to Case 21)**																				
	**C1**	**C2**	**C3**	**C4**	**C5**	**C6**	**C7**	**C8**	**C9**	**C10**	**C11**	**C12**	**C13**	**C14**	**C15**	**C16**	**C17**	**C18**	**C19**	**C20**	**C21**
Assigned group	5	13	24	7	30	15	19	10	13	18	28	10	8	25	36	6	22	36	12	33	11
Other groups	0	6	12	6	3	4	18	3	9	9	13	16	16	7	21	26	14	12	11	5	12

Seventy-two messages (11.4%) out of a total of 629 postings indicated the presence of social factors; some examples of these social cues were: “I will present the case in class…”, “Oh, I got it”, “I am not advertising for Tylenol…”, “@NicolasZhao hasn’t replied yet, but I sent email already”, etc.

Table 4 shows messages by type: the majority of student posts were “reply to comment”; only 26 @comments were posted by students. Reply to comment and @comment reflected the interaction among users, which accounted for 62.4% of the total posts.

**Table 4:** The use of direct comment, reply to comment, and @comment

**Day**	**Messages’ interactions**		
	**Direct comment**	**Reply to comment**	**@comment**
1	0	0	0
2	0	0	0
3	0	0	0
4	84	86	14
5	122	146	9
6	13	50	0
7	16	82	3
Total	235	364	26

Table 5 shows the frequency of 5 levels of cognitive skills for the 592 messages from students in MBC. The messages written by the instructor or not related to the cases (e.g. social cues) were not included. The percentages for messages of elementary clarification, in-depth clarification, and inference were 27.9%, 31.9%, and 25%, respectively. The percentage for messages of judgment and strategies were 2.5% and 2.7% respectively.

**Table 5:** The frequency of 5 levels of cognitive skills in MBC

**Reasoning skills**	**Number of messages (%)**	**Examples**
Elementary clarification	165 (27.9%)	*“Do we need to consider the withdraw symptom?”*
		*“The patient just has muscle pain.”*
In-depth clarification	189 (31.9%)	*“Both Gabapentin and Pregabalin were approved by FDA to treat DPNP…”*
		*“The patient has mild ~ moderate pain caused by exercise …ibuprofen therapy is not working well for this patient due to its short half-life.”*
Inference	148 (25%)	*“FDA has warned the suicidal risk especially for young patients, thus should let the patient know…”*
		*“The patient developed sexual dysfunction, thus should avoid SSRI…”*
		*“Need 5 weeks wash out period due to fluoxetine’s long half-life…”*
Judgment	15 (2.5%)	*“Many cold meds share the acetaminophen ingredient; if take cold meds, it’s possible to cause acetaminophen overdose…”*
		*“The priority goal is to control the pain, although Pentazocine has fewer side effects, it is not a good choice for this patient.”*
Strategies	16 (2.7%)	*“If one drug failed, it doesn’t mean other drugs in the same class would fail, we could try at least 2 SSRIs before switching to other class, I will recommend another SSRI, e.g. Sertraline 50 mg PO QD…”*
		*“Since the patient had tried Bupropion and Citalopram with full dose and duration, this is resistant, severe depression. Amitriptyline probably won’t be helpful…According to the notes, I will recommend olanzapine/fluoxetine 6 mg/25 mg PO QPM…”*

### Student attitudes of MBC

A total of 112 (89%) students completed the survey. Eighty-six percent of respondents were between 21 and 22 years of age. The MBC usage data (part of the demographic data from the questionnaire) showed that twenty-one (19%) students checked the MBC several times a day, 77 (69%) checked several times a week, 12 (11%) students used PC and never used mobile devices (smartphone, iPad, etc.) to view or post messages. The students’ attitudes toward MBC are provided in Table 6. Eight-four percent of respondents agreed that MBC helps them to express themselves better (Q1) and 79% of respondents agreed that MBC helps them understand other points of view (Q2); 81% of respondents indicated that their point of view had been acknowledged by peers and the instructor (Q3). Ninety-one percent of respondents indicated that MBC helped them to share information with others (Q4) and 56% of respondents indicated they learned from their peers (Q5). Additionally, 81% of respondents were motivated to do additional research (Q6) and 43% of respondents agreed that the collaborative learning was effective (Q7). Sixty-seven percent of respondents indicated that MBC increased their sense of community (Q8) and 66% of respondents indicated they felt comfortable participating (Q9). Fifty-six percent of respondents agreed that the amount of interaction was sufficient (Q10) and 30% of respondents agreed that the quality of interaction was good (Q11). Overall, 66% of respondents preferred MBC to traditional case studies (Q12).

**Table 6:** Students’ attitudes towards MBC (n = 112)

	**Strongly disagree, %**	**Disagree, %**	**Neutral, %**	**Agree, %**	**Strongly agree, %**
Q1: It helps me to express my opinions clearly and concisely.	0	4	13	59	25
Q2: It helps me understand other points of view.	1	4	15	50	29
Q3: My point of view has been acknowledged by my peers/teachers.	0	4	15	58	23
Q4: It helps me to share my knowledge and experience with my peers.	0	3	6	56	35
Q5: I was able to develop skills and knowledge from my peers	4	15	24	40	16
Q6: I have been stimulated to do additional readings or research.	0	5	14	43	38
Q7: Collaborative learning was effective.	4	21	32	33	10
Q8: It helps me feel connected to other students in this course.	1	7	25	44	23
Q9: I am comfortable participating, even though I am not familiar with the topics.	2	13	19	41	25
Q10: The amount of my interaction with other students was sufficient.	1	2	41	38	18
Q11: The quality of interaction with other students was good.	1	21	46	24	6
Q12: Overall using MBC was better than traditional cases study (didactic or paper-based)	3	5	26	40	26

Table 7 presents the categories, number of responses per category, and example responses for both positive and negative comments from the open-ended questions on the survey.

**Table 7:** Students’ descriptive responses regarding the MBC

**Open-ended questions**	**No of responses**
**What did you like BEST about the MBC?**	
Facilitates interaction: *“I can learn from other students’ comments…It’s easy to communicate and discuss with other students.”*	71
Allows students to communicate in an easy way: *“Team members don’t have to get together to discuss, it’s efficient and convenient…It’s easy to check online information with a simple click.”*	57
Facilitates study: *“Using the microblog made me more interested in the cases…It’s a fun assignment with clear expectations.”*	45
Allows students to express their opinions in an easy way: *“I feel more comfortable in expressing my ideas…”*	42
Facilitates team work: *“It made us work more efficiently as a team.”*	20
The activity was convenient: *“I can work on the case anywhere, anytime with my mobile devices…”*	6
**What did you like LEAST about the MBC?**	
The quality of interaction was low: *“I prefer face-to-face communication, which is passionate… The interaction is not in real-time, responses are often delayed.”*	41
The activity was inconvenient / overwhelming: *“Using the microblog was difficult and time consuming…I have to sign in to an account for the assignment.”*	33
The quality of messages was low: *“Some comments were repetitions of other students…”, “Some opinions were wrong and misleading…”*	18
The activity was distracting: *“I tended to check other messages I followed rather than the case assignment.”*	15
It was difficult to follow the stream of comments: *“There were over 50 reply comments on some cases; it was hard to check every comment on each page…”*	13

### Data analysis

The average number of comments for each student was about 5 (a total number of 592 posts for the 126 students), this demonstrated that students actively participated in the online text-based discussions using a microblog platform. It is interesting that for cases 12, 13, 16, & 21, the assigned groups had less to say regarding their own case than the other groups. One explanation for this may be that those group members were not active learners; they just posted to meet the minimum requirements and did not actively participate in the online discussion.

The social presence is low in this study because it is an online discussion with a combination of face-to-face lectures: many of them have known each other and students who use their own personal accounts (79.4%) may follow each other on microblogs already. Moreover, it’s a requirement of the course that MBC posts should be professional and related to the cases.

The ratio of “reply to comments” (364 posts) to “direct comments” (235 posts) was 1.5:1, which showed students responded more than once to direct comments on average. Additionally, the average number of posts for each case was 28, and the average ratio of “message number of assigned group” (18 posts) to “message number of other group” (11 posts) was 1.7:1, which shows that the amount of interaction among students was good.

For this MBC activity, most posts were classified as elementary, in-depth, and inference; judgement and strategy posts were seen much less frequently. This means that this MBC activity didn’t involve in-depth learning processes such as proposing a recommendation with justification, outlining the advantages and disadvantages of a therapy regimen, and making clinical judgments supported by evidence.

## Discussion

Using microblogging within an appropriate pedagogical frame can enhance classroom engagement, reflective thinking and collaborative learning to complement face-to-face discussions in the traditional classroom environment. Although microblogging has been adopted in the educational setting [5-7], this study is the first to report a case discussion format using a microblog platform to enhance pedagogy.

### Advantages

The findings from this study demonstrated some unique advantages of MBC as perceived by students. First, students indicated that MBC facilitated communication and sharing ideas. Sixty-two percent of the messages were a “reply to comment” and “@comment”, which meant that someone was replying to or addressing someone else. It could be stated that the purpose of these messages was simply communication and exchange of information. The large amount of messages that contained the sign @ promoted information sharing and interaction among students. The limitation of 140 characters required students to focus on the topic, organize their thoughts and express themselves in a concise way. Thus they found it is easy to understand other students through MBC. Secondly, students indicated that MBC increased their interest in studying the case. This may be because most of the participants were millennium students who prefer active learning using technology; and 46% of college students in China are current microblog users [29]. Therefore, case studies presented through the most popular web 2.0 application may be more attractive to pharmacy students and potentially motivated them to do additional learning. Thirdly, students agreed that MBC enhanced collaboration independent of time and place. A small number of students indicated that they could participate in the project anywhere with their smartphones (for example, in the library or classroom, or on a bus). As 67% of college students in China own smartphones and the user growth will continue at a steady double-digit pace [29], the survey results demonstrate a potential for use of mobile learning to engage students in the classroom.

### Lessons learned

The survey results suggest the MBC had some particular disadvantages as well. First, a small number of students stated that the collaborative learning was not very effective. This was probably due to a significant lag time in a few of the students’ responses resulting in a delay of one day. One way to address this deficit is to set up some guidelines beforehand, for instance a shorter discussion time interval (e.g. from 2 PM to 8 PM) to allow more effective interactions in “real-time”. Secondly, some students stated that the quality of interactions and messages was low. This might be because some comments were repetitions of classmates and some postings lacked reasoning and critical thinking skills. One way to address this deficit is to ask students to read other related comments to the case before making a new comment to avoid repetitions. Furthermore, requiring students to provide references and present a rationale for their clinical opinions will also improve the quality of the interaction. Thirdly, like other web 2.0 applications (e.g., Twitter, Facebook) [4,5], MBC is distracting: by students’ personal account, they tend to check other students’ personal posts by visiting other students’ homepage on microblog; also, they may receive messages irrelevant to the case from the people they followed. Although we recommended using a separate account, it seemed students preferred to use their own account: only 26 students (20.6%) set up a new account. Lastly, sometimes, it was hard to follow the stream of comments due to the large volume of interactions (e.g. case 13 had over 50 comments), students had to read many comments to participate.

To encourage students to fully express their ideas and opinions, the instructor only participated in online discussion when: (1) comments posted by students were misleading or wrong; (2) @comments were used to address the instructor; (3) instructor made a summary for the case after the deadline. The instructor felt comfortable with the 140 characters limit because cases for this pharmacotherapy introduction course were all small ones; besides, students had the option to add another comment if 140 characters were not enough.

For educators who would like to use MBC, there are several recommendations: (1) set up a short interval time to make the online discussion more effective; (2) encourage students to provide references or present a rationale for comments to improve the quality of interaction; (3) number each condition for a complicated case to save characters and make it easier to understand: for example, a comment starting with “R1” means recommendations for the first condition and “R2” means recommendations for the second one; (4) require students to read through other comments for a case before posting to avoid posting similar comments.

There are some limitations in our study: First, this study was based on only 1 case study and with only 1 cohort of students in a single school. Second, students were informed that a study was being conducted, thus this may have introduced the possibility of bias. Third, students could send private messages to each other on the microblog; these messages were not viewable, thus were not counted. Fourth, the cases used in this study were all simple cases for a pharmacotherapy introduction class, whether complicated cases (e.g. patient with multiple conditions) could be discussed by microblog is not tested in this study.

## Conclusions

The MBC were well received by pharmacy students in the pharmacotherapy introduction class. The majority of students agreed that MBC was an effective study tool on the aspects of information sharing, collaborative learning, promoting interaction, and sense of community. On the other hand, findings from our survey suggest that some students dislike MBC because the quality of interaction through MBC is low and it is distracting and hard to follow a large volume of the posts. Instructors planning to use MBC in their course should balance its advantages with its disadvantages.

## Appendix 1

### Depression 1: a case example

MJ is a 37-year-old male who is hospitalized for moderate depressive symptoms. This is his 3^rd^ episode of major depression in the last year. He is currently being treated with Sertraline 150 mg daily. According to his wife, he often misses 3 or 4 days of his Sertraline in a row due to forgetting his medication when traveling for his job. MJ admits that on these occasions he gets very anxious and often feels nauseated.

## Consent

Written informed consent was obtained from the patient for the publication of this report and any accompanying images.

## Abbreviations

MBC: Microblog-based case studies.

## Competing interest

The authors declare that they have no competing interest.

## Authors’ contribution

TW conceived, carried out, and drafted the manuscript. FW participated in the design of the study, analyzed the data, and revised the manuscript. Luwen Shi participated in its design and coordination. All authors read and approved the manuscript.

## Authors’ information

TW: M.Sc., Pharm.D., RPh, assistant professor of Department of Pharmacy Administration and Clinical Pharmacy, Peking University Health Science Center.

FW: M.Sc., Pharm.D., BCPS, FASHP, associate clinical professor of Pharmacy Practice, School of Pharmacy, University of Connecticut.

LS: professor and the chair of Department of Pharmacy Administration and Clinical Pharmacy, Peking University Health Science Center.

## Pre-publication history

The pre-publication history for this paper can be accessed here:

http://www.biomedcentral.com/1472-6920/13/120/prepub
